# OA15.03. Cost savings associated with mindfulness meditation and moderate exercise intervention in the common cold (The MEPARI Study)

**DOI:** 10.1186/1472-6882-12-S1-O60

**Published:** 2012-06-12

**Authors:** D Rakel, B Barrett, M Mundt, L Fortney, T Ewers

**Affiliations:** 1 University of Wisconsin School of Medicine and Public Health, Madison, USA

## Purpose

Value is defined by improving quality while reducing cost. This study's objective was to evaluate if 8-week mindfulness meditation or exercise programs can result in reduced costs related to Acute Respiratory Infections (ARIs).

## Methods

One hundred forty-nine adults ≥ 50 years were recruited from community and randomized into one of three groups: 1) wait-list observation (control), 2) meditation and 3) moderate intensity exercise. Cost associated with ARI incidence and severity was assessed by tracking self-reported medication use, and number of missed-work days and medical clinic visits, with costs per subject calculated based on average cost for generic medications, a missed-work day ($126.20) and a clinic visit ($78.70). Monte Carlo bootstrap sampling was used based on the incidence of ARI and computed to 95% confidence intervals (CI) from the sampling distributions.

## Results

The mean total cost per subject for the control group was $214 (95% CI: $105-$358), for exercise $136 (95% CI: $64-$232) and for meditation $65 (95% CI: $34-$104). The majority of the cost savings was through a reduction in missed days of work. The exercise group had the highest medication costs per subject at $16.60 compared to $5.90 for meditation (p=.004) and $7.20 (p=.046) for control. Compared to control, meditation and exercise were associated with a 70% (p=.010) and 36% (p=.334) lower cost respectively.

## Conclusion

Meditation and exercise reduce the burden of ARI and associated costs. Meditation, more then exercise, brings value to health care spending by enhancing quality of health while reducing cost for ARI. Further research is needed to show how integrative medicine therapies can add value to medical spending for other health care needs.

